# Mercury concentrations in common carp (*Cyprinus carpio*) tissues, sediment and water from fish farm along the Karoun River in Iran

**Published:** 2015-09-15

**Authors:** Payvand Maktabi, Mehran Javaheri Baboli, Ali Reza Jafarnejadi, Abolfazl Askary Sary

**Affiliations:** 1 *Young Researchers and Elite Club, Ahvaz Branch, Islamic Azad University, Ahvaz, Iran;*; 2 *Department of Fisheries,College of Agriculture and Natural Resources, Ahvaz Branch, Islamic Azad University, Ahvaz, Iran; *; 3 *Department of Soil and Water Research, Agricultural and Natural Resources Research of Khuzestan Center, Ahvaz, Iran.*

**Keywords:** *Cyprinus carpio*, Mercury, Sediment, Water

## Abstract

The Karoun River is major source of water for warm‌water fish culture industry in southwest of Iran. The aim of the present study was to investigate the distribution of mercury in tissues of marketable common carp and in bottom sediments of fish farms in Khouzestan province. This study was carried out on 45 fish farms that are located on the bank of the Karoun River in Khouzestan province, south-west Iran. Concentration of mercury (Hg) was determined using spectrophotometery in three tissues (muscles, liver and gills) of farmed common carp (*Cyprinus carpio*), water and bottom sediments of fish farms collected from three regions (North, center and south) of the Karoun River, in Khouzestan province, Iran. The concentrations of Hg in muscle tissue (2.71 mg kg^-1^ dry matter) of ﬁsh from the south were signiﬁcantly higher (*p* < 0.05) than from the other two sites. In the center and south sampling zones, Hg concentration in muscle was found to be above the maximum tolerable values provided by Food and Drug Administration standards. The Hg concentration of fish farm sediment and water samples were ranged as 0.46 to 0.48 mg kg^-1^ dry matter and 3.10 to 4.11 μg Hg L^-1^, respectively. Finally, Hg concentrations at downstream site were higher than upstream site.

## Introduction

Mercury (Hg) contamination of aquatic ecosystems is a global problem. Mercury is a toxic and hazardous metal that occurs in the aquatic environment due to natural occurrence or anthropogenic activities.^1^ Therefore, it is essential to regularly monitor any potential contamination of the environment and its impact on food chains to ensure food quality and safety.^2^^,^^3^ Fish is the last curl of the aquatic food chain, therefore, important to know the levels of pollutants can represent an ecological and human health hazard to the fish and human health.^4^^,^^5^

Mercury concentration in fish is influenced by biotic and abiotic factors such as their life cycle, life history, species feeding habits, the age and size of the fish as well as water parameters related to acidity and Hg speciation.^6^ Some fish and tissues tend to accumulate particular heavy metals and therefore must be omitted from human diets. Muscles are not always the best indicator of whole fish body contamination. Therefore, to judge fish toxicity by heavy metals, it is recommended to analyze other tissues such as liver, gills, and kidneys. Rivers are one of the best water resources for aquaculture industry, however, technology and population increasing trend have caused heavy metal contamination and reduced quality of them. The Karoun River is the largest river in Iran. It receives raw sewage from various untreated industrials and agricultural sources along its basin before entering to the Persian Gulf in Khuzestan province. The pollutions in Karoun River, involving toxic trace metals and other hazardous substances cause their bioaccumulation in fish tissues. The Mercury concentration in fish species and water of the Karoun River have been reported.^7^^,^^8^ The total area of fish farms in Iran is estimated at approximately 41458 hectare in 2009. Chinese carp (*Cyprinus carpio, Ctenopharyngodon idellus, Hypophthalmichthys molitrix, Hypophthalmichthys nobilis*) are the major warm water fishes in Iran. Khuzestan province has a high potential to produce warm fish because of water resources and climate in Iran and has represented about 21.00% of warm fish production in Iran in recent years. The aim of the present study was to investigate the distribution of mercury in tissues of marketable common carp and in bottom sediments of fish farms of Khuzestan province. 

## Materials and Methods

**Fig. 1:**
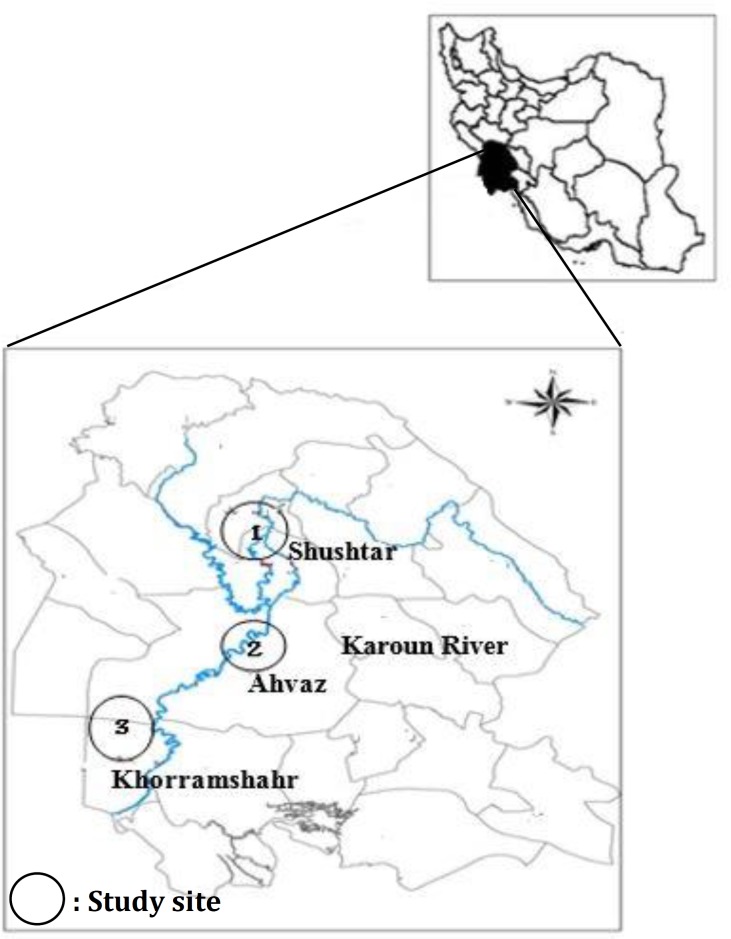
Location of study sites on Karoun River flow in Khuzestan province, Iran

**Sampling. **This study was carried out on 45 fish farms located on the bank of the Karoun River in Khuzestan province, south-west Iran in fall 2011 (Fig.1). The Karoun River flows from north to south of Khuzestan region. The fish farms are located from 30˚ 33’ to 31˚ 59’ east longitude and 48 ˚ 15’ to 48 ˚ 53’ north latitude. To compare mercury (Hg) concentrations (fish, sediment and water) on different regions of the river, three sites were sampled: Site-1 north), Site-2 (center) and Site-3 (south). This study was conducted to completely randomized design (CRD) with three treatments (i.e. sites) and three replications (three farms on each site). The concentration of Hg was measured in the muscle, gills and liver of *C. carpio*, sediment and water at three sites of the fish farms. 

**Analysis. **The samples after capture (15 fish on each farm) were placed in plastic bags with ice and transported to the laboratory. Also, sediment and water samples were taken at the same time. The bioassay samples included the total length, standard length, wide body (biometry ruler) and weight were measured by standard methods. All samples were cut into pieces and labeled, and then all sampling procedures were carried out according to internationally recognized guidelines.^7^ Fish samples were dried at 50 ˚C in oven. Afterwards samples were digested in concentrated HNO_3_ using 1 g of dried sample in 10 mL of concentrated HNO_3_ and filtered using No. 42 filter paper (Whatman, Maidstone, UK). To measure the mercury concentrations in sediment (2 m in depth), 60 mL HNO_3_ 4M to 6.90 g of dried soil was added and mixed for 12 hr at 70 ˚C temperature. The mixture was centrifuged for 15 min and filtered using the filter paper. Mercury concentrations in the extract (fish, sediment and water) were measured using inductively coupled plasma-optical emission spectroscopy (ICP-OES, JY Ultima 2C, Jobin Yvon, France).^9^ Statistical analysis was conducted with SPSS software (Version 14; SPSS Inc., Chicago, USA).

Analysis of one way variance with the Duncan multiple range test was applied to find the significant differences among means of fish tissues and sediment and water for Hg concentrations. The normality test was done by Kolmogorov-Smirnov method.

## Results

The results showed that all data were distributed as normal (*p* > 0.05). Table 1 shows the results on mean length and weight in *C. carpio* from each of the three sites of the Karoun River. The mean fish weight was numerically higher for Site-1 than other sites. But there was no statistically significant difference between the three sites for studied parameters (*p* > 0.05).

Table 2 shows the concentration of mercury (mg kg^-1^ dry matter) in gills, liver and muscles tissues at each of the three sites of the Karoun River.

Mercury concentration showed highly significant differences between organs of fish (*p* < 0.05). The mercury concentration in different tissues was ranged from 0.44 to 3.58 mg kg^-1^ dry matter.

Mercury concentration in bottom sediments and water of fish farms are shown in Table 3.

There were no significant differences observed among sites in Hg content. The lowest value was detected in bottom sediment of Site- 1 and Site-2 fish farm while the highest one in bottom sediment of Site-3 fish farm (Table 3). Content of mercury in water of fish farms under study showed no significant differences and ranged in 3.10 to 4.11 μg L^-1^. The lowest value was detected in water of Site-1 fish farm while the highest one in water of Site-3 fish farms (Table 3).

The common carp has been cultivated in all studied fish farms either in 2-year-cycle (Khuzestan province). Hg distribution pattern in tissues of *C. carpio* in Site-2 and Site-3 farms follows the order: Liver > Gill > Muscle; while this pattern in *C. carpio* in Site-1 farm follows the order: Gill > Liver > Muscle (Table 4).

**Table 1:** Mean of carp bioassay on the studied sites of Karoun River. Data are presented as mean ± SE.

**Region**	**Weight (kg)**	**Total length (cm)**	**Standard length (cm)**	**Body width (cm)**
**Site-1 (North)**	1.28 ± 0.16	40.27 ± 0.16	32.96 ± 1.49	13.4 ± 0.66
**Site-2 (Center)**	1.26 ± 0.05	40.03 ± 0.63	33.86 ± 0.49	14.06 ± 0.21
**Site-3 (South)**	1.09 ± 0.07	37.16 ± 0.93	31.80 ± 0.79	13.86 ± 0.41

There are no significant differences between the data in each column (*p* > 0.05).

**Table 2:** Concentrations of mercury (mg kg^-1^ dry matter) in gills, liver and muscles tissues at each of the three sites of the Karoun River. Data are presented as mean ± SE.

**Tissues**	**Site-1 (north)**	**Site-2 (center)**	**Site-3 (south)**
**Gill**	3.02 ± 0.05 b	3.19 ± 0.03 a	3.02 ± 0.06 b
**Liver**	2.96 ± 0.12	3.22 ± 0.28	3.58 ± 0.19
**Muscle**	0.44 ± 0.06 b	2.55 ± 0.51 a	2.71 ± 0.06 a

ab Different superscripts indicate significant difference in each row (*p* < 0.05).

**Table 3:** Concentrations of mercury in sediment (mg Hg kg^-1^ dry matter) and water (μg L^−1^) at each of the three sites of the Karoun River. Data are presented as mean ± SE.

**Water/Sediment**	**Site-1 (north)**	**Site-2 (center)**	**Site-3 (south)**
**Sediment **	0.46 ± 0.04	0.46 ± 0.07	0.48 ± 0.00
**Water **	3.10 ± 0.20	3.58 ± 0.04	4.11 ± 0.14

There are no significant differences between the data in each row (*p* > 0.05).

**Table 4:** Organ-wise distributions of mercury concentrations in *C. carpio*

**Tissues**	**References**
**Muscle > Liver > Gills**	Čelechovská *et al*.^10^
**Muscle > Kidney > Liver **	Svobodová *et al*.^11^
**Muscle > Kidney > Liver**	Has-Schon *et al*.^12^
**Gills > Liver > Muscles**	Site-1 in this study
**Liver > Gill > Muscle**	Site-2 and Site-3 in this study

## Discussion

Knowledge of heavy metal concentrations such as Hg in fish is important with respect to nature of management and human consumption of fish. The Hg concentrations in sediment, water and fish tissues are used as the main indicators of loading the fish farm environment. The concentrations of mercury were found generally higher in the liver and gills than muscle tissues. Usually, liver is more often known as an indicator of water pollution than any other organs in fish. The high accumulation of metals in the liver may be related to the fact that the liver plays an important role in accumulation and detoxification.^13^^,^^14^

The distribution of Hg in muscles and internal organs of fish depends, among other things, on the degree of contamination of the environment.^15^^,^^16^ In polluted locations, Hg concentrations in internal organs are usually significantly higher than its concentrations in muscle.^15^^,^^17^ Hg distribution in lightly contaminated localities seems to take the following pattern: Muscle > Kidney > Liver > Gonads.^18^^,^^19^

The Hg permissible limit proposed by the FDA is 1 mg kg^-1^ dry matter.^20^ In the central and southern parts of sampling sites, the concentrations of Hg in the muscle (edible part) of *C. carpio* were higher than the FDA,^20^ however, in the north site the concentrations of Hg in the muscle were lower than FDA level.^20^

Mercury concentrations were much higher in fish samples than those of water because of its active absorption and accumulation in fish tissues. Results of the previous study showed that the mean concentration of Hg in the muscle of *B. grypus* and *B. xanthopterus* in the Karoun River were 0.73 mg kg^-1^ dry matter and 1.28 mg kg^-1^ dry matter, respectively.^7^

The concentrations of total Hg were detected in freshwater fish collected from different farms around the Pearl River Delta, PR China ranged from 7.43 ± 1.87 to 76.70 ± 13.60 ng g^-1 ^wet weight.^21^

Changes in the process of absorption and accumulation of Hg concentrations in fish can be divided into exogenous, characteristics of the water body, and endogenous factors, characteristic of the individuals or species. Exogenous exposures include pH, sulfur and organic matter, hardness of water environment, temperature. Endogenous exposures include species, habitat and food preferences, metabolic rate, age, growth rate, size, mass, and diet.^22^ In this study, increasing Hg concentrations trend was due to common carp mainly fed with barley or dry-pellet fish feed. Low-quality barley and pellet feed may be possible sources of Hg contamination. Also, making a comparison on the pollution load of the industries located at these three sites indicated that a quantity of 56502 ton per year organic load and 260186 ton per year mineral load from various industries were discharged into the Karoun River.^8^ The Karoun basin with an annual load of 5.40 tons, takes the most heavy metals pollution load in Khouzestan province.^8^

As expected, the pollutant levels were generally higher downstream than upstream due to decreasing human activities were recognized near the upstream site. Hg concentration in dry matter of pond bottom sediments was performed in south and west Bohemia (range: 0.03 to 0.35 mg kg^-1^ dry matter).^23^ Another study has also demonstrated Hg in dry matter of bottom sediment and water of the farms various regions in Hungary ranged 11.80 to15.70 mg kg^-1^ dry matter and less than 0.2 mg L^-1^, respectively.^24^ The criterion recommended for protection of human and aquatic health by international organization and all the values is higher than 0.001 mg L^-1^.^25^ For freshwater ecosystems, the probable effect concentration for sediment Hg concentration is 1.06 μg g^-1^.^26^ The Hg concentration observed in fish farms may be explained by the highly developed manufacturing industry and the use of contaminated feed and disinfectants causing increased concentrations of total Hg in fish farm sediments.^27^ The Hg methylation in sediment was affected by many factors, organic matter and sulfate concentration, farm age and pH.^28^

Finally, based on the results, it seems that the general increasing trend of mercury concentrations in tissue, water and sediment from upstream to downstream parts of the Karoun River was probably related to the increased domestic sewage, industrial waste and agricultural activities. However, the high levels of mercury concentrations, is needed to minimize fish consumptions and daily intake of the permissible mercury limits.
